# Global frequency, diagnosis, and treatment of hereditary angioedema with normal C1 inhibitor

**DOI:** 10.1016/j.jacig.2025.100446

**Published:** 2025-02-27

**Authors:** Markus Magerl, Marc A. Riedl, Luisa Karla Arruda, Andrea Bauer, Alejandro Berardi, Jonathan A. Bernstein, Laurence Bouillet, Matthew Buckland, Thomas Buttgereit, Danny M. Cohn, Timothy Craig, Roberta F. Criado, Aurélie Du-Thanh, Olivier Fain, Margarida Gonçalo, Jens Greve, Anete Sevciovic Grumach, Mar Guilarte, Constance Katelaris, Tamar Kinaciyan, Elena A. Latysheva, Ramon Lleonart, Oscar Calderón Llosa, Eli Mansour, Vesna Grivcheva-Panovska, Claudio Parisi, Nelson Augusto Rosario Filho, Amélia Spínola Santos, Petra Staubach, Anna Valerieva, Solange Oliveira Rodrigues Valle, Sherry Danese, Julie Ulloa, Paul K. Audhya, Marcus Maurer

**Affiliations:** a Angioedema Center of Reference and Excellence (ACARE), Institute of Allergology, Charité–Universitätsmedizin Berlin, corporate member of Freie Universität Berlin and Humboldt-Universität zu Berlin, Berlin, Germany; b Fraunhofer Institute for Translational Medicine and Pharmacology ITMP, Allergology and Immunology, Berlin, Germany; c Division of Allergy and Immunology, University of California—San Diego, La Jolla, Calif; d Clinical Hospital of Ribeirão Preto Medical School, University of São Paulo, São Paulo, Brazil; e Department of Dermatology, University Allergy Center, University Hospital Carl Gustav Carus, Technical University Dresden, Dresden, Germany; f Instituto de Asma, Alergia y Enfermedades Respiratorias, Corrientes, Argentina; g University of Cincinnati College of Medicine, Division of Rheumatology, Allergy and Immunology and Bernstein Allergy Group and Clinical Research Center, Cincinnati, Ohio; h French National Reference Center for Angioedema (CREAK), Grenoble Alpes University, Grenoble, France; i Barts Health NHS Trust, London, United Kingdom; j Amsterdam UMC Location AMC, Amsterdam, The Netherlands; k Penn State University, University Park, Pa; l Alergo Skin, Santo André, Brazil; m Montpellier University Hospital, Montpelier, France; n Sorbonne Université, service de médecine interne, AP-HP, Hôpital St Antoine, Paris, France; o Clínica de Dermatologia, University Hospital, Coimbra Local Health Unit, and Faculty of Medicine, University of Coimbra, Coimbra, Portugal; p Department of Otorhinolaryngology, Head and Neck Surgery, Ulm University Medical Center, Ulm, Germany; q Clinical Immunology, University Center Faculty of Medicine ABC, Santo André, Brazil; r Allergy Department, Hereditary Angioedema Reference Center (CSUR-74), Vall d’Hebron Research Unit (VHIR), Hospital Universitari Vall d’Hebron, Barcelona, Spain; s Campbelltown Hospital and Western Sydney University, Campbelltown, Australia; t Department of Dermatology, ACARE, Medical University of Vienna, Vienna, Austria; u Pirogov Russian National Research Medical University, Moscow, Russia; v Hospital Universitari de Bellvitge, Barcelona, Spain; w SANNA–Clínica el Golf, San Isidro, Peru; x Universidade Estadual de Campinas (UNICAMP), Campinas, Brazil; y ACARE North Macedonia, PHI University Clinic of Dermatology, Ss Cyril and Methodius Skopje University, Skopje, Republic of North Macedonia; z Hospital Italiano de Buenos Aires, Buenos Aires, Argentina; aa Federal University of Parana, Curitiba, Brazil; bb ACARE, Hereditary Angioedema Unit, Immunoallergology Department, Unidade Local de Saúde de Santa Maria, Faculdade de Medicina da Universidade de Lisboa, Lisbon, Portugal; cc Hautklinik und Poliklinik Universitätsmedizin der Johannes Gutenberg-Universität Mainz, Mainz, Germany; dd Department of Allergology, Medical University of Sofia, University Hospital “Alexandrovska,” Sofia, Bulgaria; ee Clementino Fraga Filho University Hospital Federal University of Rio de Janeiro, Rio de Janeiro, Brazil; ff Outcomes Insights, Agoura Hills, Calif; gg KalVista Pharmaceuticals Inc, Cambridge, Mass

**Keywords:** Hereditary angioedema, normal C1INH, prevalence, diagnostics, treatment, management

## Abstract

**Background:**

Hereditary angioedema (HAE) is a rare genetic disease, most frequently associated with deficiency or dysfunction in the C1 inhibitor protein. HAE with normal C1 inhibitor (HAE-nC1INH) lacks standardized diagnostic tests, limiting precise prevalence estimates and development of specific treatment guidelines.

**Objective:**

This study sought to describe the global frequency, diagnostic pathway, and current treatment patterns of HAE-nC1INH.

**Methods:**

Board-certified HAE-treating physicians from accredited Angioedema Centers of Reference and Excellence (ACAREs) were invited to complete a 27-item online survey between December 2022 and April 2023.

**Results:**

Thirty physicians from 30 ACAREs across 15 countries reported a mean of 71 (range, 11-148) patients with HAE assessed/treated within the previous 12 months. On average, physicians estimated 24% (range, 2-44%) of patients with HAE were diagnosed with HAE-nC1INH, most of whom were adults (88%). To diagnose HAE-nC1INH, physicians most commonly assessed family history and plasma C4 levels (90% each), and C1 function and quantitative levels (87% each). On-demand and prophylactic treatment patterns varied widely across countries, with an average (range) of 56% (33-100%) of patients receiving on-demand treatment only, and 37% (0-67%) receiving both on-demand and prophylactic treatment. Physicians identified the greatest unmet needs in HAE-nC1INH management as treatment specifically indicated for this patient population and availability of an oral treatment.

**Conclusion:**

HAE-nC1INH may be more prevalent than previously reported. Importantly, our findings revealed varying diagnostic and treatment approaches. Validated, accessible diagnostic biomarkers and clinical outcomes derived from rigorous clinical trials assessing mechanistically based treatments would advance understanding and management of HAE-nC1INH.

Hereditary angioedema (HAE) is a rare disease characterized by painful, debilitating, and unpredictable attacks of tissue swelling in various locations of the body, including the skin, abdomen, and upper respiratory tract.^1^^,^^2^ Patients with HAE experience a substantial burden related to disruptions in daily life, direct and indirect costs, increased risk of comorbidities and adverse consequences,^3^, ^4^, ^5^ and potentially life-threatening laryngeal attacks, which can occur at any age, including in children.^6^

There are several forms of genetically identifiable HAE. HAE Type 1 (HAE-C1INH-Type1) is the most common form (approximately 85% of cases) and presents with C1 inhibitor (C1INH) deficiency, while HAE Type 2 (HAE-C1INH-Type2) is caused by dysfunctional C1INH.^2^^,^^7^ Both forms are autosomal-dominant conditions caused by mutations in *SERPING1,* with a combined estimated prevalence of 1 in 50,000.^1^ Other cases of HAE, though phenotypically similar, present with normal C1INH activity (HAE-nC1INH).^2^^,^^8^ HAE-nC1INH currently includes 6 recognized types that are based on genetically identifiable mutations in genes for factor XII (HAE-FXII), angiopoietin-1 (HAE-ANGPT), plasminogen (HAE-PLG), kininogen 1 (HAE-KNG), myoferlin (HAE-MYOF), and heparan sulfate-glucosamine 3-*O*-sulfotransferase 6 (HAE-HSST),^2^ plus two recently described variants in *CPN1* and *DAB2IP*.^9^^,^^10^ However, most patients diagnosed with HAE-nC1INH have no identifiable gene mutation.^2^ HAE-nC1INH is reported to be less common than HAE-C1INH Types 1 and 2; however, precise prevalence rates of HAE-nC1INH are lacking.^11^

Current guidelines for the diagnosis and management of HAE largely focus on HAE-C1INH Types 1 and 2, while recommendations specific to HAE-nC1INH have not been fully developed.^2^ This is because of the challenges associated with confirming a diagnosis of HAE-nC1INH, as standardized, validated biochemical diagnostic tests are lacking, and genetic tests for the fraction of cases with known disease-causing mutations are not widely available.^2^ The 2021 World Allergy Organization/European Academy of Allergy and Clinical Immunology guidelines recommended the following for differential diagnosis of HAE-nC1INH: suspicion of HAE should prompt laboratory testing to assess C1INH function, C1INH protein levels, and C4 levels; and patients who are suspected to have HAE and have normal C1INH levels and function should undergo genetic testing for known mutations underlying HAE-nC1INH.^2^ Additionally, these guidelines state that diagnostic procedures (eg, genetic testing) should be used where available and that other options should be considered where recommended procedures are not available. The guidelines note that family history is an important tool for identifying patients with HAE-nC1INH.^2^ Unlike HAE-C1INH Types 1 and 2, treatment of HAE-nC1INH has not been established by randomized, placebo-controlled trials.^1^

A recent survey of real-world practice provided estimates of HAE-nC1INH prevalence and described diagnoses and management strategies in the United States leveraging claims data^12^; however, these may not be globally representative. To address this gap, HAE-treating physicians in certified Angioedema Centers of Reference and Excellence (ACAREs)^13^ located in multiple countries were surveyed online. The objectives of this study were: (1) to assess the global frequency of HAE-nC1INH; (2) to describe the diagnostic pathway of patients with HAE-nC1INH, such as referral patterns, testing, and time to diagnosis; and (3) to explore current patterns for on-demand and prophylactic treatment of HAE-nC1INH. Additionally, physicians provided insights into the unmet needs regarding management of people with HAE-nC1INH.

## Methods

### Data collection

This was a voluntary online survey of HAE-treating physicians representing angioedema specialists in certified ACAREs. These accredited angioedema referral centers fulfill robust requirements based on the experience of the Global Allergy and Asthma European Network (GA^2^ LEN) and input from patients, general practitioners, and specialists.^13^ The survey consisted of 27 multiple-choice questions, rank-order questions, and scale-based responses using a symmetrical 7-point Likert scale of agreement with presented statements ranging from “extremely dissatisfied” to “extremely satisfied.” The survey questions are available in this article’s Online Repository available at www.jaci-global.org. In a follow-up survey, the primary criteria for diagnosis were assessed to better understand how physicians diagnose HAE-nC1INH.

Potential participating physicians were identified from accredited ACAREs with the longest tenure as ACAREs. Physicians were contacted and screened to determine whether they met the following inclusion criteria: physicians who had completed specialty training (eg, board certified) and had treated at least one patient with HAE-nC1INH within the past 12 months.

One eligible physician per ACARE was invited to take part in the online survey between December 2022 and April 2023 via a secure electronic data-capture system. The survey was provided in English and took approximately 15 minutes to complete. Responses were based on physician recall. Before initiating the survey, all participating physicians provided informed consent for their data to be used anonymously or in aggregate. The study was declared exempt from review by an institutional review board.

### Data analysis

Analysis of the survey data was performed using descriptive statistics. Continuous variables were summarized as means, medians, and ranges, whereas categorical variables were summarized as counts and percentages.

## Results

### Survey participants and findings

The survey was completed by 30 physicians from 30 ACAREs in 15 countries (Table I). The follow-up survey was completed by all but 2 physicians from 2 ACAREs.

**Table I:** Characteristics of 30 physician-respondents

Characteristic	ACARE, no. (%)
Country	
Brazil	6 (20.0)
Germany	4 (13.3)
France	3 (10.0)
United States	3 (10.0)
Argentina	2 (6.7)
Portugal	2 (6.7)
Spain	2 (6.7)
Australia	1 (3.3)
Austria	1 (3.3)
Bulgaria	1 (3.3)
Netherlands	1 (3.3)
North Macedonia	1 (3.3)
Peru	1 (3.3)
Russia	1 (3.3)
United Kingdom	1 (3.3)
Specialty	
Allergy and/or immunology	19 (63.3)
Dermatology	7 (23.3)
Internal medicine	3 (10.0)
Otolaryngology	1 (3.3)
Years in practice, mean (range)	25 (6-45)

**Fig 1:**
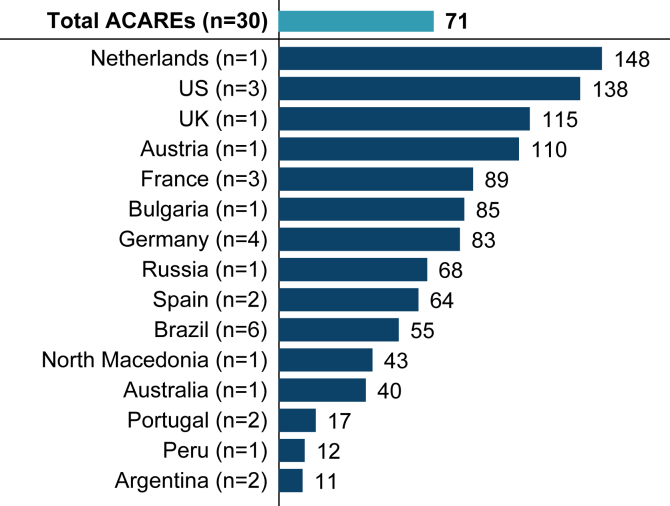
Average HAE patient volume over previous 12 months. Average number of patients per center treated for any type of HAE (confirmed or suspected); n represents number of centers per country.

Over the previous 12 months, on average, ACAREs treated 71 patients with HAE (including HAE-C1INH-Type1, HAE-C1INH-Type2, and HAE-nC1INH). There was wide variability across countries, ranging from a minimum of 11 patients in Argentina to 148 patients in the Netherlands (Fig 1). Twelve ACAREs provided estimates of treated patients with HAE subtypes. Among these ACAREs, an estimated 763 (40-83 per center) patients with HAE-C1INH-Type1, 139 (0-25 per center) patients with HAE-C1INH-Type2, and 298 (2-55 per center) patients with HAE-nC1INH were reported (see Table E1 in the Online Repository available at www.jaci-global.org).

### HAE-nC1INH frequency estimates

**Fig 2:**
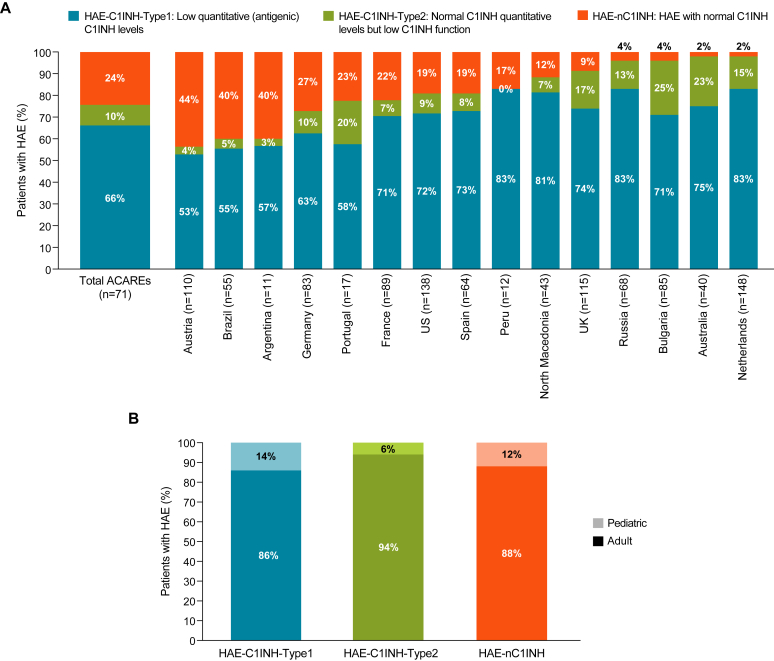
Estimates of (**A**) HAE subtypes by country and (**B**) HAE subtypes among adult and pediatric patients. *(A)* n represents average HAE patient volumes per center over last 12 months. *(B)* Pediatric patients were age <18 years.

On average, nearly one quarter (24%) of patients with HAE at ACAREs were diagnosed with HAE-nC1INH (Fig 2, *A*); the proportion of patients with HAE-nC1INH ranged from 2% in Australia and the Netherlands to 44% in Austria. Across ACAREs and HAE subtypes, most patients (≥86%) were adults (Fig 2, *B*). Six countries (Australia, Austria, Bulgaria, France, the Netherlands, and North Macedonia) reported no pediatric (age <18 years) patients with a presumptive diagnosis of HAE-nC1INH.

### HAE-nC1INH diagnosis-related findings

**Fig 3:**
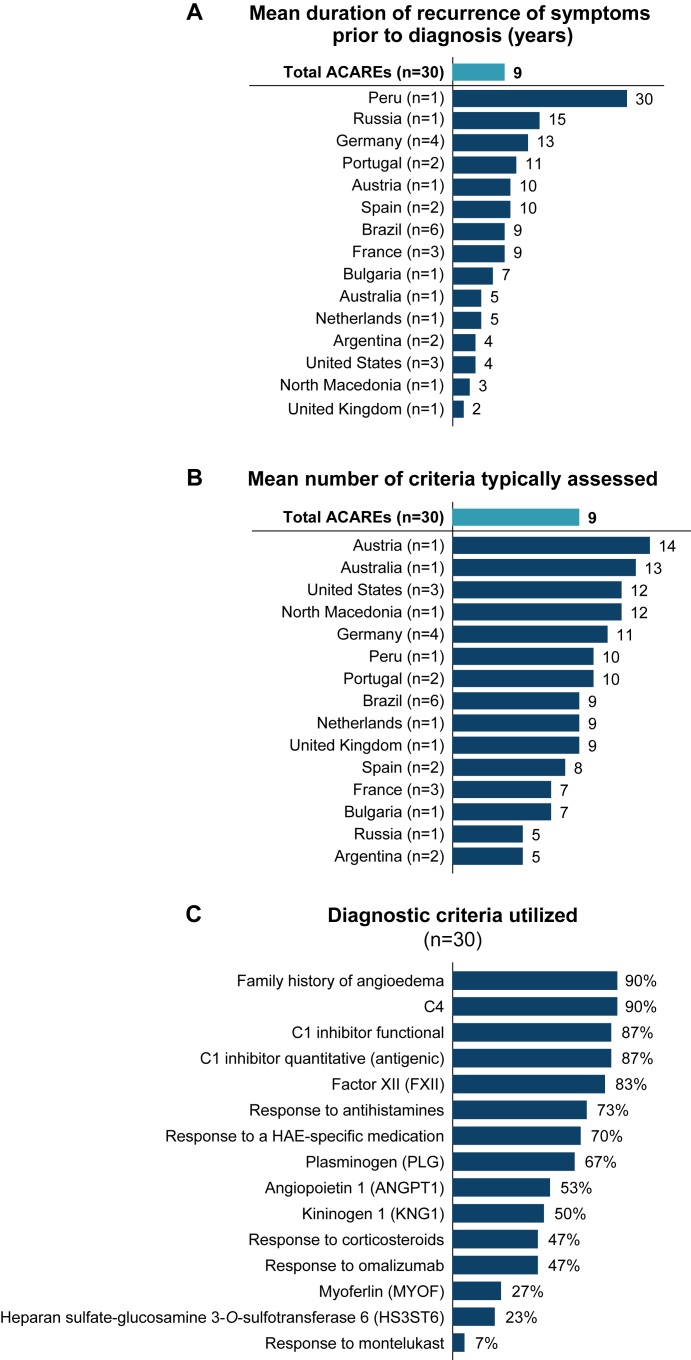
Diagnosis of HAE-nC1INH. **(A)** Duration of recurrence of symptoms before diagnosis, **(B)** number of criteria typically assessed, and **(C)** diagnostic criteria utilized. *(A)* Time to diagnosis was defined as time from which symptoms were experienced to receiving clinical diagnosis. *(C)* Physicians were asked to select which criteria they typically assess to make diagnosis of HAE-nC1INH (multiple selections allowed).

The average time to diagnosis of HAE-nC1INH (defined as the time from which symptoms were first experienced to the time at which a conclusive diagnosis was received) across ACAREs was 9 years, ranging widely from 2 years in the United Kingdom to 30 years in Peru (Fig 3, *A*). When physicians were asked to report the minimum and maximum length of time to HAE-nC1INH diagnosis observed in their personal experience, there was a wide range reported within countries—for example, 2-20 years in the United States, 1-20 years in Portugal, 3-23 years in Brazil, 4-29 years in France, 2-30 years in Peru, and 2-31 years in Germany.

ACARE physicians reported assessing an average of 9 (range, 5-14) different criteria when diagnosing HAE-nC1INH (Fig 3, *B*). The most common criteria used to diagnose HAE-nC1INH included the assessment of a positive family history of angioedema (90%), normal plasma C4 levels (90%), normal C1INH functional and quantitative (antigenic) levels (each 87%), mutations in factor XII (83%), lack of response to antihistamines (73%), response to HAE-specific medications (70%), and mutations in plasminogen (67%) (Fig 3, *C*). Utilization of genetic testing (other than factor XII) for diagnosis was highly variable across countries, and was the primary criterion used to confirm diagnosis of HAE-nC1INH in ∼50% of ACAREs (see Fig E1, *A,* in the Online Repository available at www.jaci-global.org). Icatibant (91%) and intravenous plasma-derived C1INH (10-52%) were the most common treatments for confirming diagnosis (see Fig E2, also in the Online Repository). Other criteria to confirm diagnoses were used in 13% of ACAREs, with a positive family history noted in 6 of 8 free-text responses (Fig E1, *B*).

### HAE-nC1INH treatment patterns

**Fig 4:**
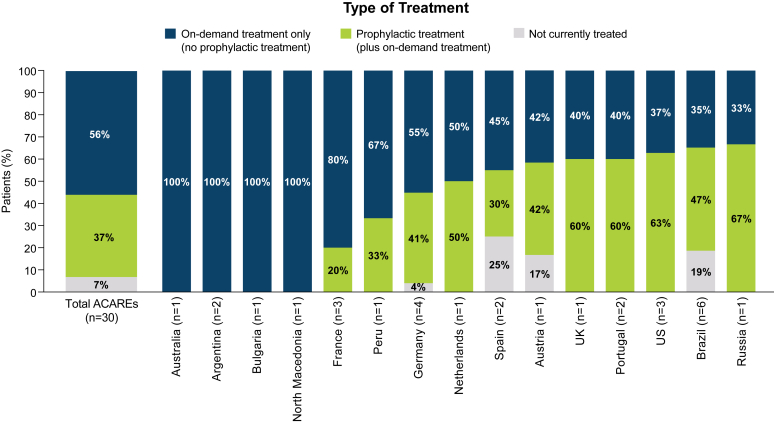
Management of patients with HAE-nC1INH. Physicians were asked to select the type of treatment prescribed for their patients with presumptive HAE-nC1INH diagnosis.

ACAREs reported that, on average, 56% of patients received on-demand treatment only, with 4 individual centers reporting that all their patients with HAE-nC1INH received on-demand treatment only. Over one-third (37%) of patients received prophylactic plus on-demand treatment, ranging from 0 to 67% depending on country (Fig 4). On average, 7% of patients were reported to have received no treatment.

#### On-demand treatment

**Fig 5:**
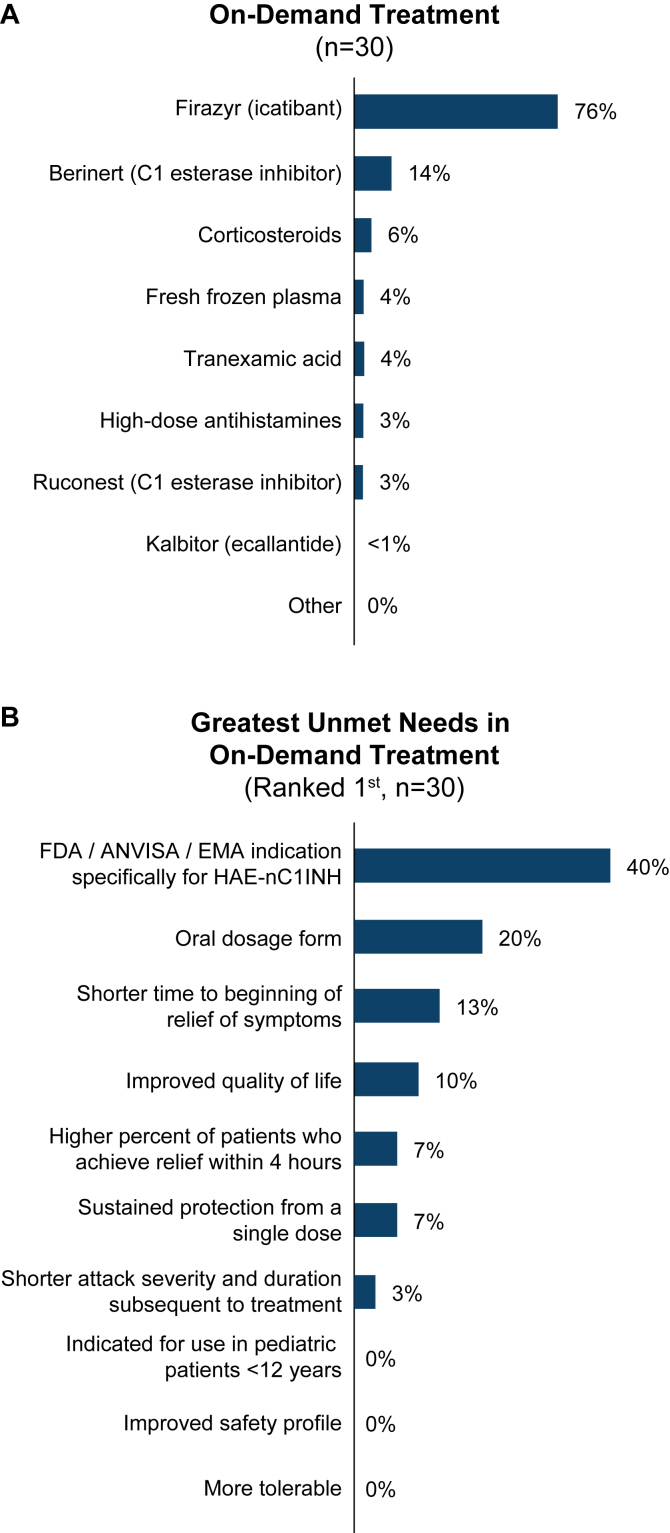
(**A**) Most common on-demand treatments and (**B**) greatest unmet needs in on-demand treatment. *(A)* Physicians indicated how many patients with HAE-nC1INH were currently receiving each on-demand treatment. *(B)* Physicians selected ≤3 unmet needs in order of importance. *ANVISA,* Agência Nacional de Vigilância Sanitária; *EMA,* European Medicines Agency; *FDA,* US Food and Drug Administration.

Icatibant was the most commonly prescribed on-demand treatment for HAE-nC1INH attacks, followed by intravenous plasma-derived C1INH (Fig 5, *A*). Patients receiving on-demand treatment experienced an average of 6 (range, 1-30) attacks per year.

#### Prophylactic treatment

**Fig 6:**
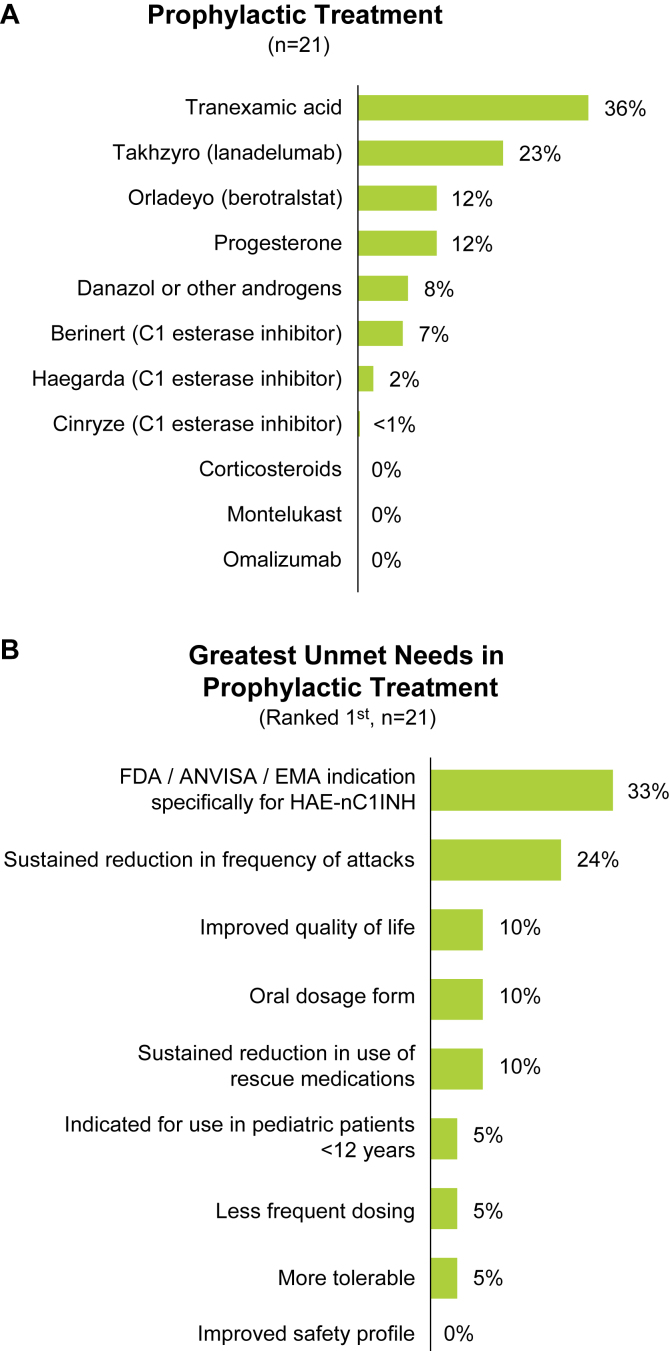
(**A**) Most common prophylactic treatment and (**B**) unmet needs in prophylactic treatment. *(A)* Physicians indicated how many patients with HAE-nC1INH were currently prescribed each prophylactic treatment. *(B)* Physicians selected ≤3 unmet needs in order of importance. *ANVISA,* Agência Nacional de Vigilância Sanitária; *EMA,* European Medicines Agency; *FDA,* US Food and Drug Administration.

Attack frequency (43%) and severity (38%) were identified as the top 2 drivers for initiating prophylactic treatment for HAE-nC1INH, followed by patient request (5%), knowledge of triggers for attack (5%), and Angioedema Control Test scores (5%). ACAREs recommended prophylactic treatment for patients who had, on average, 12 (range, 3-34) or more attacks per year. Before taking prophylactic treatment, patients had, on average, 19 (range, 6-50) attacks per year. Across ACAREs, tranexamic acid (36%) and lanadelumab (23%) were the most common prophylactic treatments for HAE-nC1INH, followed by berotralstat (Fig 6, *A*).

#### Unmet treatment needs

ACARE providers identified an HAE-nC1INH–specific indication as the greatest unmet need in on-demand treatment for HAE-nC1INH (Fig 5, *B*). In free-text responses, physicians emphasized a need for improved diagnostic criteria and treatment that is faster acting and in oral/tablet formulation (see the Online Repository available at www.jaci-global.org).

Similar to on-demand treatment, on average, 33% of ACARE providers identified an HAE-nC1INH–specific indication as the greatest unmet need in prophylactic treatment for HAE-nC1INH (Fig 6, *B*). This was followed by 24% of providers indicating a need for prophylactic treatment to provide sustained reduction in the frequency of attacks. Free-text responses from physicians are provided in the Online Repository.

## Discussion

This is the first study to survey HAE-treating physicians across multiple countries about the frequency and treatment patterns of patients with HAE-nC1INH. On average, 24% of patients with HAE at ACAREs were diagnosed with HAE-nC1INH, demonstrating a potentially larger population of patients with HAE-nC1INH within clinical practices than previously reported in the literature (16-23%).^12^ However, this rate varied widely across countries, from 2% to 9% of patients with HAE in the Netherlands, Australia, Bulgaria, Russia, and the United Kingdom, and up to 27% to 44% of patients with HAE in Germany, Argentina, Brazil, and Austria. Whether this disparity is due to regional genetic differences, the variable diagnostic criteria utilized, or other factors is unknown.^14^ There was a disparity in the proportion of pediatric (12%) to adult (88%) patients with HAE-nC1INH, potentially related to reliance on clinical symptoms or a differing natural history/age at symptom onset for HAE-nC1INH compared to HAE-C1INH Types 1 or 2. For example, age at symptom onset is reportedly higher among patients with HAE-nC1INH (age ≥20 years in HAE-FXII, HAE-PLG, and HAE-KNG) compared with HAE-C1INH-Type1/2 (childhood/adolescence), where female patients with HAE-FXII especially experience attacks triggered by estrogens.^2^^,^^15^ Previous studies similarly reported HAE-nC1INH is less commonly diagnosed in children, with a mean age at symptom onset at 27 years, and only 8% of patients experiencing clinical onset before the age of 10 years.^16^ A slightly higher proportion (∼10-14%) of patients with HAE-nC1INH due to mutations in *F12* (ie, the gene encoding coagulation FXII) reported symptoms before the age of 12 years, potentially as a result of the hormonal influence of puberty.^1^^,^^2^

Our findings highlight the challenges in the diagnosis of HAE-nC1INH. Consistent with findings in patients with HAE-C1INH-Type1 and HAE-C1INH-Type2, a significant delay in diagnosis (as long as 30 years) was reported in patients with HAE-nC1INH.^3^^,^^5^^,^^17^ The most common diagnostic criteria for HAE-nC1INH were a positive family history, normal C4 assessment, normal C1INH functional and quantitative (antigenic) assessment, lack of response to antihistamines (ie, H_1_ antagonists), response to HAE-specific medications, and mutations in factor XII and plasminogen. Overall, these diagnostic assessments included assessment of family history, C1INH functional and quantitative levels, C4 levels, and genetic testing, which aligns with current international guidelines for diagnosis of HAE-nC1INH,^2^ although the primary diagnostic criteria varied across countries, with some reporting that 100% of HAE-nC1INH diagnoses were primarily confirmed using genetic testing (North Macedonia and Spain), while others primarily used response to treatment for 100% of HAE-nC1INH patient diagnoses (the Netherlands, Bulgaria, and Peru) despite also assessing relevant genetic variants or family history. The variability of these results by country shows an uneven geographical distribution of HAE-nC1INH that is difficult to fully explain. Differences in prevalence of certain genetic variants (ie, founder effects) in some countries or regions is an important consideration, but it is likely that the implementation of different criteria (ie, lack of a standardized approach) for the diagnosis of HAE-nC1INH leads to differences in frequency. The combination of inconsistent weighting of criteria and the lack of broad availability of genetic testing are likely among the greatest drivers of the observed variability. Genetic testing for HAE-nC1INH is not only inaccessible in some regions but is also limited by a small number of known pathogenic variants found in only the subset of patients with phenotypic symptoms of HAE-nC1INH.^2^^,^^18^ These limitations warrant a diagnostic approach that combines genetic tests with assessment of other biomarkers and does not rely heavily on family history or treatment responses. An in-depth family history is challenging to gather in clinical practice and can be unreliable or inaccurate as a result of numerous factors: recall bias, unknown or incorrect information (eg, due to estrangement, adoption, or paternal discrepancy), possible *de novo* mutations, and variable penetrance resulting in phenotypic variation or asymptomatic carriers.^19^ Responses to on-demand treatment may be a poor diagnostic indicator as a result of subjective assessment, patient response bias associated with open-label treatment, or failing to more definitively exclude mast cell–mediated angioedema with adequate treatment such as omalizumab therapy.^20^ While there are currently no validated biochemical markers to identify individuals with HAE-nC1INH, novel assays for assessing C1INH function and measurement of kinins or stimulated kallikrein activity (eg, dextran sulfate, cold induced) may potentially fulfill the crucial need to identify and diagnose HAE-nC1INH.^1^^,^^21^ Additionally, the detection of bradykinin degradation products may be more clinically practical than plasma bradykinin measurement, which is technically challenging, given the extremely short half-life of bradykinin.^18^

The ACARE clinicians who were surveyed use a variety of approaches to treat patients with HAE-nC1INH. Patients with HAE-nC1INH often received drugs indicated for management of patients with HAE-C1INH Types 1 or 2, including icatibant for on-demand treatment and lanadelumab for prophylactic treatment. On average, physicians recommended prophylactic treatment after 12 attacks per year for patients with HAE-nC1INH, although this recommendation varied widely across countries (range, 3-34 attacks per year). These findings are consistent with case series and observational studies that indicate use of HAE-C1INH-Type1/2–indicated treatment options in some individuals with HAE-nC1INH^4^^,^^17^; however, robust treatment efficacy and safety data specific to HAE-nC1INH are still lacking. Notably, the only randomized, placebo-controlled clinical trial of treatment of HAE-nC1INH completed to date evaluated efficacy and safety of lanadelumab for prevention of attacks (NCT04206605).^22^ A reduction in plasma kallikrein and cleaved high-molecular-weight kininogen activity was observed, with inhibition being numerically higher in the lanadelumab group compared with placebo; however, the study did not meet its primary end point, finding no significant difference in the number of angioedema attacks between groups.^22^

Clinicians identified the greatest unmet need as treatments indicated specifically for HAE-nC1INH, with some physicians noting a need for therapies that are effective across each subtype of HAE-nC1INH. Physicians also indicated a need for oral on-demand and prophylactic treatments, consistent with preferences reported by patients with HAE currently receiving prophylactic intravenous and subcutaneous therapies.^23^ Currently, the oral treatments available for HAE prophylaxis are berotralstat,^24^ attenuated androgens (not recommended for first-line treatment in evidence-based HAE management guidelines),^2^ and tranexamic acid (off-label use for HAE).^2^ A positive phase 3 trial was recently reported for the investigational oral plasma kallikrein inhibitor, sebetralstat, for the on-demand treatment of HAE attacks in patients with HAE-C1INH-Type1/2 (NCT05259917).^25^

The following limitations should be considered when interpreting these findings. These data depend on physician recall, and there was no requirement to validate against patient charts. Information on how medical records were maintained was not collected. Because the diagnostic approach for HAE is most often by exclusion, whether physicians excluded other potential diagnoses was taken into account but not explicitly requested. Additionally, the data do not necessarily reflect the overall situation at individual ACAREs but are limited to the patients cared for by the participating doctor. The inability to confirm each HAE-nC1INH diagnosis with a standard diagnostic biomarker or genetic test introduces the potential for some patients to be missed and others erroneously included. It is important for future studies to investigate the proportions of patients with confirmed HAE-nC1INH mutations. Furthermore, while participating physicians had, on average, practiced for 25 (range, 6-45) years, information on how many years of experience physicians had specifically managing patients with more common types of HAE (ie, HAE-C1INH Types 1 and 2) was not collected. The patients seen at ACAREs are a highly selected HAE population and HAE frequency may be overestimated compared to those seen in other clinics.

Current literature on HAE-nC1INH diagnosis and treatment is limited, with ongoing development of expert consensus on diagnosis of HAE-nC1INH. These findings may be valuable for future study development, potentially improving diagnosis and management of patients with HAE-nC1INH.

## Disclosure statement

Sponsored and funded by 10.13039/100031004KalVista Pharmaceuticals and conducted in collaboration with ACARE. KalVista participated in the design and conduct of the study. Statistical analyses were conducted by Outcome Insights, funded by 10.13039/100031004KalVista. The initial draft of the report was prepared by a medical writer funded by KalVista. Subsequent revisions and the final decision to submit the report for publication were made by the authors, who vouch for the accuracy and completeness of the data and for the fidelity of the study to the protocol.

Data-sharing statement: KalVista Pharmaceuticals accepts requests from qualified researchers who wish to access clinical trial data and associated information, such as Clinical Study Reports (CSRs) with appropriately redacted appendices to protect participant privacy. Inquiries may be directed to DSP@kalvista.com.

Disclosure of potential conflict of interest: M. Magerl received personal fees/nonfinancial support from Astria, Shire/Takeda, CSL Behring, Pharming, BioCryst, KalVista Pharmaceuticals, Pharvaris, Ionis, Intellia, and Octapharma. M. Riedl is or recently was a speaker and/or advisor for and/or has received research funding from Astria, BioCryst, BioMarin, Celldex, CSL Behring, Cycle Pharma, Grifols, Intellia, Ionis, KalVista, Pfizer, Pharming, Pharvaris, Sanofi-Regeneron, and Takeda. A. Bauer received personal fees and/or other support from BioCryst, CSL Behring, and Takeda. J. A. Bernstein has received grants and/or honoraria from KalVista, BioCryst, BioMarin, CSL Behring, Intellia, Ionis, Pharming, Pharvaris, and Shire/Takeda; and serves as immediate past president of the American Academy of Allergy, Asthma & Immunology. L. Bouillet has consulted/served as speaker for, engaged in research and educational projects with, and/or accepted travel grants from BioCryst, CSL Behring, Takeda, KalVista, Pharvaris, and Intellia. M. S. Buckland is or recently was a speaker and/or advisor for and/or has received research funding from BioCryst, CSL Behring, Pharming, and Takeda. T. Buttgereit is or recently was a speaker and/or advisor for and/or has received research funding from Aquestive, BioCryst, CSL Behring, GSK, Hexal, KalVista, Medac, Novartis, Pharming, Pharvaris, Roche, Sanofi-Aventis, Swixx BioPharma, and Takeda. D. M. Cohn has received consulting fees paid to the institution, honoraria paid to the institution, meeting/travel support, research support, and/or served on advisory boards from KalVista, Astria, BioCryst, CSL Behring, Intellia, Ionis Pharmaceuticals, Pharming, Pharvaris, and Takeda; and serves a leadership role in the HAE International medical advisory panel for Central Eastern Europe and Benelux. T. Craig reports research with BioMarin, KalVista, Pharvaris, GSK, CSL Behring, Takeda, Ionis, Intellia, Astra, Pfizer, Regeneron and Grifols; speaker for Takeda, CSL Behring, and Grifols; and consultant for BioMarin, Intellia, CSL Behring, Takeda, BioMarin, Ionis, Astra, KalVista, and BioCryst; has Center designations from the International Hereditary Angioedema Association and Alpha-1 Foundation; and is a member of the medical advisory board for the HAE-A. R. F. Criado has received grants, personal fees, and/or nonfinancial support from Amgen, Lilly, Novartis, and Pfizer. A. Du-Thanh has received meeting/travel support, research support, and/or served on advisory boards for BioCryst, Novartis, Pharvaris, and Takeda. O. Fain has received personal fees from Behring, BioCryst, KalVista, and Takeda; and a grant from BioCryst. M. Gonçalo has participated as speaker/advisor and/or received department research funding from AbbVie, Almirall, AstraZeneca, Elli-Lilly, LEO Pharmaceutica, Pfizer, Sanofi, Novartis, and Takeda. J. Greve is or recently was a speaker and/or advisor for and/or has received research funding from BioCryst, CSL Behring, KalVista, and Takeda. A. S. Grumach receives research funding from the Brazilian National Council for Scientific and Technological Development (CNPq) and a grant of researcher initiative from Shire/Takeda; and is or recently was a speaker and/or advisor for Catalyst Pharmaceuticals, CSL Behring, Takeda, KalVista, Pharvaris, Pint-Pharma, and MultiCare. M. Guilarte is or recently was a speaker and/or advisor for and/or has received research funding from BioCryst, CSL Behring, KalVista, Pharvaris, and Takeda. C. Katelaris has received personal fees from AstraZeneca, CSL Behring, GSK, KalVista, Pharvaris, and Sanofi. T. Kinaciyan is or recently was a speaker and/or advisor for and/or has received research funding from BioCryst, CSL Behring, KalVista, KINIKSA, Novartis, Pharvaris, Sanofi-Aventis, and Takeda. E. A. Latysheva has received personal fees from CSL Behring, Octapharma, Novartis, and Takeda; and a grant from Takeda. R. Lleonart has received consulting fees, honoraria, payment for expert testimony, meeting/travel support, and/or served on advisory boards for KalVista, BioCryst, CSL Behring, Pharming, and Takeda. E. Mansour has received grants and/or personal fees from AstraZeneca, CSL Behring, GSK, Novartis, Sanofi, and Takeda. V. Grivcheva-Panovska has served on advisory boards for CSL Behring, Takeda, BioCryst, KalVista, and Pharvaris; and has received honoraria for presentations from CSL Behring and Takeda, an unrestricted research grant from CSL Behring, and travel assistance from CSL Behring. A. Spínola Santos has received personal fees from BioCryst, CSL Behring, Diater Laboratorio de Diagnóstico y Aplicaciones SA, and Takeda. P. Staubach has received grants and/or consulting fees from Novartis, CSL Behring, Sanofi, Takeda, KalVista, BioCryst, and Pharvaris. A. Valerieva has received honoraria for educational lectures, acted as consultant for, and/or has received sponsorship for educational meetings and/or research projects from Shire/Takeda, Pharming Group NV, CSL Behring, SOBI, AstraZeneca, Berlin-Chemie/Menarini Group, Teva, Novartis, Ewopharma, Stellergenes-Greer, Pharvaris, KalVista, Ionis, Astria, and Organon. S. Danese and J. Ulloa received consulting fees from KalVista. P. K. Audhya is an employee of KalVista. M. Maurer was a speaker and/or advisor for and/or received research funding from Astria, BioCryst, CSL Behring, Intellia, Ionis, KalVista, Pharvaris, and Takeda. The rest of the authors declare that they have no relevant conflicts of interest.
