# Supplementary material for: Global frequency, diagnosis, and treatment of hereditary angioedema with normal C1 inhibitor
Source: J Allergy Clin Immunol Glob. 2025 Feb 27;4(3):100446. doi: 10.1016/j.jacig.2025.100446 (PMC12020835; doi:10.1016/j.jacig.2025.100446)
Supplement: Supplementary Material [file mmc1.docx]

# Supplemental Materials

## Physician survey

### Screener

1. [Randomize] What is your primary medical specialty? (Select one)
   1. Allergy and/or immunology
   2. Otolaryngology
   3. Dermatology
   4. Internal Medicine
   5. Other (please specify) ____

1. Are you board-certified in this specialty? (Select one)
   1. Yes
   2. No
2. For how many years have you been practicing in this specialty? (Specify)
   1. ____ years

3a. What is the name of your ACARE center with which you are affiliated? (Select one)

1. <https://acare-network.com/centers/>
2. In the last 12 months, how many patients have you personally treated for any type of hereditary angioedema (confirmed or suspected)? (Specify)
   1. _____

[Terminate if Q5=0 and exit screener]

1. Of your [Q5] hereditary angioedema patients, how many are diagnosed with: (Specify for each row)

| 1. Hereditary Angioedema Type 1 (HAE-1):   Low quantitative (antigenic) C1-INH levels |  |
| --- | --- |
| 1. Hereditary Angioedema Type 2 (HAE-2):   Normal C1-INH quantitative levels but low C1-INH function |  |
| 1. Hereditary Angioedema with Normal C1-Inhibitor (HAE-normal C1-INH): HAE with normal C1-INH | [Terminate if 0] |
| Total | [Must sum to Q5] |

### Informed Consent

*We are pleased to share that you qualify to participate in this survey. We would like to inform you that:*

- Taking part in this survey is voluntary.
- If you choose to participate, you may exit the survey and return to complete it another time. If you change your mind and do not wish to participate, you may exit the survey at any time.
- Personal information that could potentially identify you will NOT be requested or collected.
- If you fully complete the survey, you will be offered an honorarium as outlined in your email invitation. Your email will not be stored or saved for any other purpose.

Do you wish to participate in this research? If so, please indicate your consent. (Select one)

- Yes, I consent to participate
- No, I do not wish to participate 🡪 Exit survey

*Congratulations, you qualify to participate in this research. Please click the button below to continue.*

### Survey

1. For each sub-type of hereditary angioedema, how many of your patients are pediatric (< 18 years) and how many are adults? (Specify)

| [If Q6a>0] | # Pediatric | # Adult |
| --- | --- | --- |
| 1. Hereditary Angioedema Type 1 (HAE-1): Low quantitative (antigenic) C1-INH levels |  |  |

[Must sum Q6a]

| [If Q6b>0] | # Pediatric | # Adult |
| --- | --- | --- |
| 1. Hereditary Angioedema Type 2 (HAE-2): Normal C1-INH quantitative levels but low C1-INH function |  |  |

[Must sum Q6b]

|  | # Pediatric | # Adult |
| --- | --- | --- |
| 1. Hereditary Angioedema with Normal C1-Inhibitor (HAE-nC1-INH*)*: HAE with normal C1-INH |  |  |

[Must sum Q6c]

1. [Randomize] To make a diagnosis of HAE-nC1-INH, which of the following do you typically assess? (Select all that apply)
   1. C1-inhibitor quantitative (antigenic)
   2. C1-inhibitor functional
   3. C4
   4. Plasminogen (PLG)
   5. Angiopoietin 1(ANGPT1)
   6. Factor XII (FXII)
   7. Kininogen 1 (KNG1)
2. Myoferlin (MYOF)
3. Heparan sulfate-glucosamine 3-O-sulfotransferase 6 (HS3ST6)
4. Response to antihistamines
5. Response to montelukast
6. Response to omalizumab
7. Response to corticosteroids
8. Response to a HAE-specific medication
9. Family history of angioedema
10. Other (specify) ____

[From Re-contact survey] Please think about your patients diagnosed with HAE-normal C1-INH. We recognize that oftentimes both genetic testing and response to therapy are used to make the final diagnosis. To the best of your recollection what percentage of patients were **primarily confirmed** using each of the following: (Specify for each row)

| 1. Genetic testing was used as the primary criteria to confirm nC1 |  |
| --- | --- |
| 1. Response to approved HAE-specific medication and/or lack of response to mast cell targeted treatment was the primary criteria to confirm nC1 |  |
| 1. Criteria other than genetic testing and response to medications was the primary criteria |  |
| Total | 100% |

[From Re-contact survey] [If c > 0%] What was the primary criterion used to confirm nC1 in this [Q3_c]% of patients? [Unaided]

_______________________________

1. [If Q8_l. Randomize] Which of the following HAE-specific medications do you use to inform your diagnosis of HAE-nC1-INH (e.g., response to medication suggests diagnosis of HAE-nC1-INH)? (Select all that apply)
   1. Cinryze (C1 esterase inhibitor)
   2. Haegarda (C1 esterase inhibitor)
   3. Takhzyro (lanadelumab)
   4. Berinert (C1 esterase inhibitor)
   5. Ruconest (C1 esterase inhibitor)
   6. Firazyr (icatibant)
   7. Kalbitor (ecallantide)
   8. Orladeyo (berotralstat)
   9. Other (specify)
   10. None of the above
2. On average, for how many years do HAE-nC1-INH patients experience symptoms prior to receiving a conclusive diagnosis (e.g., have a delay in diagnosis)? If less than 1 year, please enter “1” below. (Specify)
3. ______ years
4. Based upon your personal experience, what is the minimum and maximum length of time (in years) a HAE-nC1-INH patient experiences symptoms prior to receiving a conclusive diagnosis? If less than 1 year, please enter “1.” (Specify)

|  | Years |
| --- | --- |
| Minimum |  |
| Maximum |  |

*Next, we will explore your treatment approach for your HAE-nC1-INH patients.*

1. How many of your [Q6c] HAE-nC1-INH patient(s) are currently taking: (Specify)

| Acute treatment only (no preventative treatment) |  |
| --- | --- |
| Preventative treatment (plus acute treatment for attacks as necessary) |  |
| Not currently treated |  |
| Total | [Must sum to Q6c] |

1. [If Q12 Preventative >0] [Randomize] What drives your decision to recommend preventative therapy for a HAE-nC1-INH patient? Please select your top 3 motivating factors in order of importance. (Select top 3. Capture order of importance)
   1. Attack frequency
   2. Attack severity
   3. Predictability (or patterns) of attacks
   4. Knowledge of triggers for attack
   5. Patient request
   6. Favorable reimbursement for preventative treatment
   7. Angioedema Control Test (AECT)
   8. Other (specify) ______
2. [If Q13a selected] On average, how many attacks per year would motivate you to recommend preventative treatment for a HAE-nC1-INH patient? (Specify)
   1. _____ attacks per year
3. [Q12_preventative treatment > 0] Please consider your HAE-nC1-INH patient(s) who are currently taking a preventative treatment. On average, how many attacks per year did these patients have **prior to preventative treatment**? (Specify)
4. ____ attacks per year
5. [Q13_acute treatment only > 0%] Please consider your HAE-nC1-INH patient(s) who are **not** taking a preventative treatment (acute treatment only). On average, how many attacks per year do these patients have? (Specify)
6. ____ attacks per year

*The next section of the survey focuses on treatment of acute attacks of HAE-nC1-INH patients.*

1. [Randomize. Must sum to at least 100%] What percent of your treated HAE-nC1-INH patient(s) are currently taking each of the following for treatment of **acute attacks**? Your answers do not need to sum to a specific value. (Specify)

| 1. Berinert (C1 esterase inhibitor) |  |
| --- | --- |
| 1. Ruconest (C1 esterase inhibitor) |  |
| 1. Firazyr (icatibant) |  |
| 1. Kalbitor (ecallantide) |  |
| 1. High-dose antihistamines |  |
| 1. Fresh Frozen Plasma |  |
| 1. Corticosteroids |  |
| 1. Other (please specify) ____ |  |

1. In addition to the treatments listed above, what other medications do you prescribe for the treatment of **acute attacks** in your HAE-nC1-INH patients? (Unaided) If you do not prescribe any other medications, continue to the next question.
   1. ______
   2. ______
   3. ______
   4. ______
   5. ______
2. How satisfied are you with each of these **acute treatment options** your HAE-nC1-INH patient(s) are currently taking in terms of:
   1. Time to beginning of relief of symptoms

|  | Extremely Dissatisfied |  |  |  |  |  | Extremely Satisfied |
| --- | --- | --- | --- | --- | --- | --- | --- |
| [insert options where Q17 > 0] | 1 | 2 | 3 | 4 | 5 | 6 | 7 |
|  | 1 | 2 | 3 | 4 | 5 | 6 | 7 |
| … |  |  |  |  |  |  |  |
|  |  |  |  |  |  |  |  |

- 1. Percent of patients who feel much better within 4 hours

|  | Extremely Dissatisfied |  |  |  |  |  | Extremely Satisfied |
| --- | --- | --- | --- | --- | --- | --- | --- |
| [insert options where Q17 > 0] | 1 | 2 | 3 | 4 | 5 | 6 | 7 |
|  | 1 | 2 | 3 | 4 | 5 | 6 | 7 |
| … |  |  |  |  |  |  |  |
|  |  |  |  |  |  |  |  |

- 1. Ability to reduce severity and duration of attack

|  | Extremely Dissatisfied |  |  |  |  |  | Extremely Satisfied |
| --- | --- | --- | --- | --- | --- | --- | --- |
| [insert options where Q17 > 0] | 1 | 2 | 3 | 4 | 5 | 6 | 7 |
|  | 1 | 2 | 3 | 4 | 5 | 6 | 7 |
| … |  |  |  |  |  |  |  |
|  |  |  |  |  |  |  |  |

- 1. Sustained protection after one dose

|  | Extremely Dissatisfied |  |  |  |  |  | Extremely Satisfied |
| --- | --- | --- | --- | --- | --- | --- | --- |
| [insert options where Q17 > 0] | 1 | 2 | 3 | 4 | 5 | 6 | 7 |
|  | 1 | 2 | 3 | 4 | 5 | 6 | 7 |
| … |  |  |  |  |  |  |  |
|  |  |  |  |  |  |  |  |

- 1. Route of administration

|  | Extremely Dissatisfied |  |  |  |  |  | Extremely Satisfied |
| --- | --- | --- | --- | --- | --- | --- | --- |
| [insert options where Q17 > 0] | 1 | 2 | 3 | 4 | 5 | 6 | 7 |
|  | 1 | 2 | 3 | 4 | 5 | 6 | 7 |
| … |  |  |  |  |  |  |  |
|  |  |  |  |  |  |  |  |

- 1. Tolerability

|  | Extremely Dissatisfied |  |  |  |  |  | Extremely Satisfied |
| --- | --- | --- | --- | --- | --- | --- | --- |
| [insert options where Q17 > 0] | 1 | 2 | 3 | 4 | 5 | 6 | 7 |
|  | 1 | 2 | 3 | 4 | 5 | 6 | 7 |
| … |  |  |  |  |  |  |  |
|  |  |  |  |  |  |  |  |

1. Please describe your greatest unmet needs for the acute treatment of attacks in patients with HAE-nC1-INH? (Unaided)
2. ______________________
3. [Randomize] Of the following, which are your greatest **unmet needs** for the **acute treatment** of attacks in patients with HAE-nC1-INH? Please select up to 3 in order of importance. (Select up to 3. Capture order of importance. You may use the directional arrows to change your selections or order of importance.)
4. EMA [Brazil: ANVISA] indication specifically for treatment of patients with HAE-nC1-INH
5. Oral dosage form
6. Shorter time to beginning of relief of symptoms
7. Shorter attack severity and duration subsequent to treatment
8. Higher percent of patients who achieve relief within 4 hours
9. Sustained protection from a single dose
10. More tolerable
11. Improved safety profile
12. Indicated for use in pediatric patients <12 years
13. Improved quality of life
14. Other (specify) ____

[If Q12 Preventative >0.] *Now, we will focus on use of preventative treatments for HAE-nC1-INH patients.*

1. [Randomize] What percent of your HAE-nC1-INH patient(s) taking a preventative treatment are receiving each of the following for **prevention of attacks**? Your answers must sum to 100%. (Specify)

| 1. Cinryze (C1 esterase inhibitor) |  |
| --- | --- |
| 1. Haegarda (C1 esterase inhibitor) |  |
| 1. Takhzyro (lanadelumab) |  |
| 1. Berinert (C1 esterase inhibitor) |  |
| 1. Tranexamic acid |  |
| 1. Progesterone |  |
| 1. Danazol or other androgens |  |
| 1. Corticosteroids |  |
| 1. Montelukast |  |
| 1. Omalizumab |  |
| 1. Orladeyo (berotralstat) |  |
| 1. Other (please specify) ___ |  |
| Total | 100% |

1. In addition to the treatments listed above, what other medications do you prescribe for the **prevention** of attacks in your HAE-nC1-INH patients? (Unaided) If you do not prescribe any other medications, continue to the next question.
   1. ______
   2. ______
   3. ______
   4. ______
   5. ______
2. How satisfied are you with each of these **preventative treatment options** for your HAE-nC1-INH patient(s) in terms of:
3. Reduction in HAE attacks

|  | Extremely Dissatisfied |  |  |  |  |  | Extremely Satisfied |
| --- | --- | --- | --- | --- | --- | --- | --- |
| [insert options where Q22 > 0] | 1 | 2 | 3 | 4 | 5 | 6 | 7 |
|  | 1 | 2 | 3 | 4 | 5 | 6 | 7 |
| … |  |  |  |  |  |  |  |
|  |  |  |  |  |  |  |  |

1. Reduction in use of rescue medications

|  | Extremely Dissatisfied |  |  |  |  |  | Extremely Satisfied |
| --- | --- | --- | --- | --- | --- | --- | --- |
| [insert options where Q22 > 0] | 1 | 2 | 3 | 4 | 5 | 6 | 7 |
|  | 1 | 2 | 3 | 4 | 5 | 6 | 7 |
| … |  |  |  |  |  |  |  |
|  |  |  |  |  |  |  |  |

1. Route of administration

|  | Extremely Dissatisfied |  |  |  |  |  | Extremely Satisfied |
| --- | --- | --- | --- | --- | --- | --- | --- |
| [insert options where Q22 > 0] | 1 | 2 | 3 | 4 | 5 | 6 | 7 |
|  | 1 | 2 | 3 | 4 | 5 | 6 | 7 |
| … |  |  |  |  |  |  |  |
|  |  |  |  |  |  |  |  |

1. Tolerability

|  | Extremely Dissatisfied |  |  |  |  |  | Extremely Satisfied |
| --- | --- | --- | --- | --- | --- | --- | --- |
| [insert options where Q22 > 0] | 1 | 2 | 3 | 4 | 5 | 6 | 7 |
|  | 1 | 2 | 3 | 4 | 5 | 6 | 7 |
| … |  |  |  |  |  |  |  |
|  |  |  |  |  |  |  |  |

1. Please describe your greatest **unmet needs** in the **preventative treatment** of HAE-nC1-INH patients? (Unaided)
2. ______________________
3. [Randomize] Of the following, which are your greatest unmet needs in the preventative treatment of HAE-nC1-INH patients? Please select up to 3 in order of importance. (Select up to 3. Capture order of importance. You may use the directional arrows to change your selections or order of importance.)
4. EMA [Brazil: ANVISA] indication specifically for preventative treatment of patients with HAE-nC1-INH
5. Sustained reduction in frequency of attacks
6. Sustained reduction in use of rescue medications
7. Less frequent dosing
8. Oral dosage form
9. More tolerable
10. Improved safety profile
11. Indicated for use in pediatric patients <12 years
12. Improved quality of life
13. Other (specify) ____

## Unmet needs in on-demand treatment

*Argentina*

“There is great difficulty in accessing medication without a genetic diagnosis. In our country it is only feasible and with difficulty, the diagnosis of the FXII mutation.”

“Our main problem is the difficulty in accessing medication. Ideally, I think it would be good to have both the plasmatic C1 inhibitor and icatibant for each patient. On the other hand, the possibility of having another type of administration for the C1 inhibitor would facilitate self-application.”

*Australia*

“Access to drug as needed. We rely on compassionate access as treatment is not available for this indication in our country.”

*Austria*

“Effective oral treatments of attacks are needed in any type of HAE.”

*Brazil*

“Oral medication.”

“Lack of accessibility.”

“Some patients do not respond to the mentioned therapies.”

“Availability of HAE specific medications in my country.”

“Cost effective oral medication.”

“In my country, any C1 esterase replacement isn’t covered by Health insurance. It is necessary for general doctors and ER doctors to have more knowledge about the disease.”

*Bulgaria*

“As therapies are not licensed for treatment officially, patients feel insecure about treatment of the first episodes (they tend to try antihistamines and corticosteroids first and wait, possibly compromising bradykinin targeted therapies).”

*France*

“Oral treatment instead of injection.”

“Lack of oral and/or subcutaneous treatment for pregnant women and/or studies showing the safety of Icatibant during pregnancy.”

“Women with F12 gene mutation are symptomatic during pregnancy and Icatibant is contraindicated: an alternative treatment would be welcome. Firazyr is not tolerable in terms of pain during the injection.”

*Germany*

“Oral tablet which is rapidly acting within 15 min.”

“Oral therapy.”

“Drug for oral therapy.”

“Approved medication.”

*Netherlands*

“Fast acting oral drug!”

*North Macedonia*

“Easy to administer, not too painful, efficient and reliable medications.”

*Peru*

“Here in Perú, we have tranexamic acid for prophylactic treatment, and high doses of tranexamic acid for acute attacks, we use Ecallantide only when is laryngeal attack, is our last option, and the patients bought the medication before the laboratory cancel the registry in our country.”

*Portugal*

“Need guidelines and specific treatments for HAE- nC1INH.”

“My two patients are not having severe acute attacks since they are on danazol. Only one once tried icatibant in a moderate attack and it was not all that effective (not comparable to patients with HAE-C1-INH).”

*Spain*

“The main problem is that most patients are asymptomatic, or they present with attacks very infrequently. But, at least in my experience, few patients have attacks out of estrogenic influence.”

*Russia*

“One injection is not always enough.”

*United Kingdom*

“Effective therapy that is licensed in this patient group.”

*United States*

“Diagnostic test.”

“Understanding MOA of this condition to have more targeted therapy. Many patients use a lot of on-demand therapy - more than HAE patients.”

“1. Reliable access to rescue medication (difficulty getting payor authorization) 2. Route of administration not idea (IV or painful SQ for some pts).”

## Unmet needs in prophylactic treatment

*Austria*

“Oral treatments with high efficacy and tolerability more than 90 % of patients.”

*Brazil*

“There is no consensus or guidelines developed. We use real-life experience only.”

“Access to more specific treatments.”

“High cost of the preventative treatments, limits availability.”

“The biological treatments are not covered by health insurance.”

*France*

“Subcutaneous injection.”

“Effective and easy to use treatment, suitable for pregnant women.”

*Germany*

“More licensed drugs, orally and SC.”

“Oral therapy for acute attacks in addition to prophylactic.”

“Approved medication for patients younger than 12 years.”

*Netherlands*

“Long-acting drug or curative therapy.”

*Portugal*

“Need specific treatment and easy administration.”

“A more efficient oral preventive treatment without the adverse events of oral androgens and oral treatment for acute moderate to severe attacks.”

*Russia*

“More effective and safe”

*Spain*

“No licensed drugs; clear indications/criteria; blocking bradykinin must be useful for those HAE-nC1INH with mutations related or affecting KKS, but what about ANGP1? MYOF? Heparan?”

“There isn’t any drug approved.”

“Need target therapies for specific MOA.”

“Oral medication with improved side effect profile and improved consistent efficacy. More predictable consistent efficacy across the population - I find the variability in treatment response to be much.”

“Something that works.”

*United Kingdom*

“Licensed available therapies that work for all nC1-HAE.”

## Table SI. Number of patients with HAE subtypes receiving treatment at ACAREs

| **ACARE** | **HAE-C1INH-Type1, n** | **HAE-C1INH-Type2, n** | **HAE-nC1INH, n** |
| --- | --- | --- | --- |
| Campbelltown Public Hospital, Australia | 75 | 23 | 2 |
| Amsterdam UMC Location AMC, Netherlands | 83 | 15 | 2 |
| University Hospital "Alexandrovska", Medical University of Sofia, Bulgaria | 71 | 25 | 4 |
| NRC Institute of lmmunology FMBA Russia, Russia | 83 | 13 | 4 |
| SANNA ─ Clínica el Golf, Peru | 83 | 0 | 17 |
| Klinik für Dermatologie, Venerologie und Allergologie, Charité – Universitätsmedizin Berlin, Berlin, Germany | 80 | 0 | 20 |
| Occupational Dermatology Department of Dermatology, University Hospital Carl Gustav Carus, Technical University Dresden, Dresden, Germany | 45 | 25 | 30 |
| Hospital Vall d'Hebron, Spain | 58 | 10 | 32 |
| Hautklinik und Poliklinik Universitätsmedizin der Johannes Gutenberg-Universität Mainz, Mainz, Germany | 50 | 10 | 40 |
| University of California, San Diego, United States | 50 | 10 | 40 |
| University Center Health ABC, Brazil | 45 | 3 | 52 |
| Complexo Hospital de Clinicas Universidade, Brazil | 40 | 5 | 55 |
| **Total** | **763** | **139** | **298** |

Physicians were asked to indicate how many of their patients with HAE were diagnosed with (specify for each): HAE-C1INH-Type1 (low quantitative [antigenic] C1INH levels), HAE-C1INH-Type2 (normal C1INH quantitative levels but low C1INH function), and HAE-nC1INH (HAE with normal C1INH). Of the 28 physicians who completed the follow-up survey, 12 reported values for this question.

ACARE, Angioedema Center of Reference and Excellence; C1INH, C1 inhibitor; HAE, hereditary angioedema; HAE-C1INH-Type1, HAE with low quantitative (antigenic) C1INH levels; HAE-C1INH-Type2, normal quantitative C1INH levels but low C1INH function; HAE-nC1INH, HAE with normal C1INH activity.

## Figure S1. Primary criteria for confirmation of HAE-nC1INH diagnosis


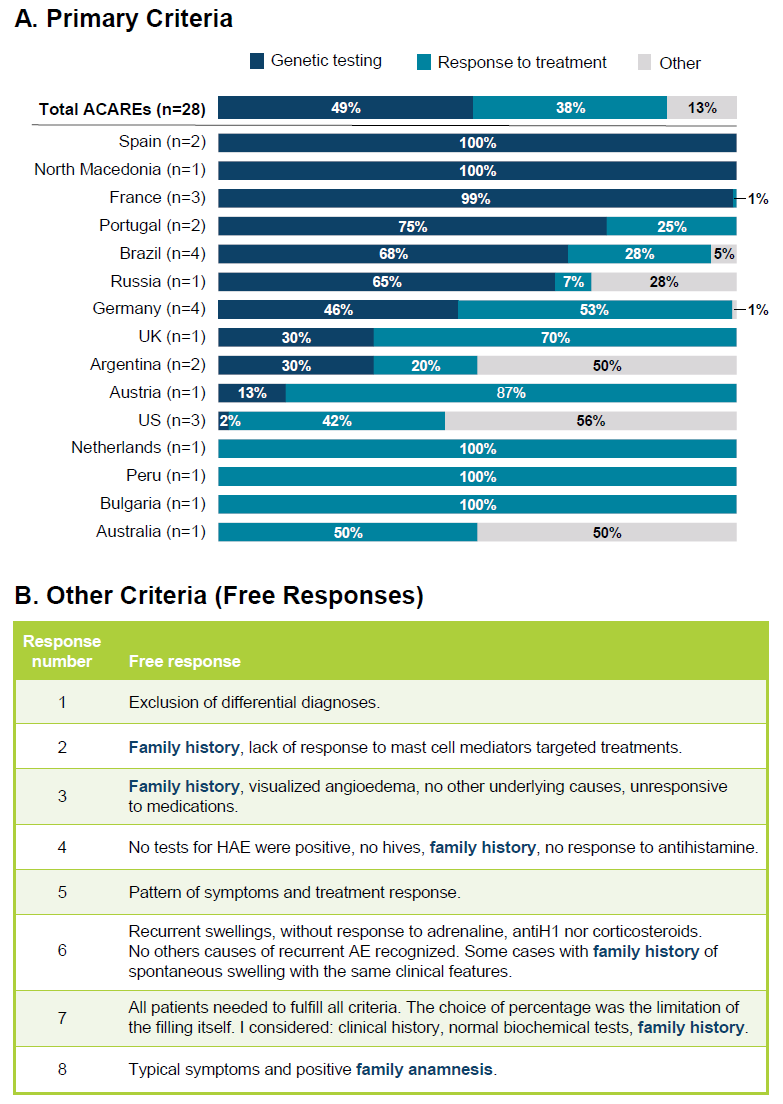


Panel A: Physicians were asked, “To the best of your recollection what percentage of patients were primarily confirmed using each of the following,” and asked to select from a list of criteria. The follow-up survey was completed by 28/30 physicians. Panel B: Physicians were asked, for those patients confirmed using criteria other than genetic testing and response to treatment, “What was the primary criterion used to confirm nC1?”

ACARE, Angioedema Center of Reference and Excellence; HAE, hereditary angioedema.

## Figure S2. Treatment responses assessed to inform diagnosis of HAE-nC1INH


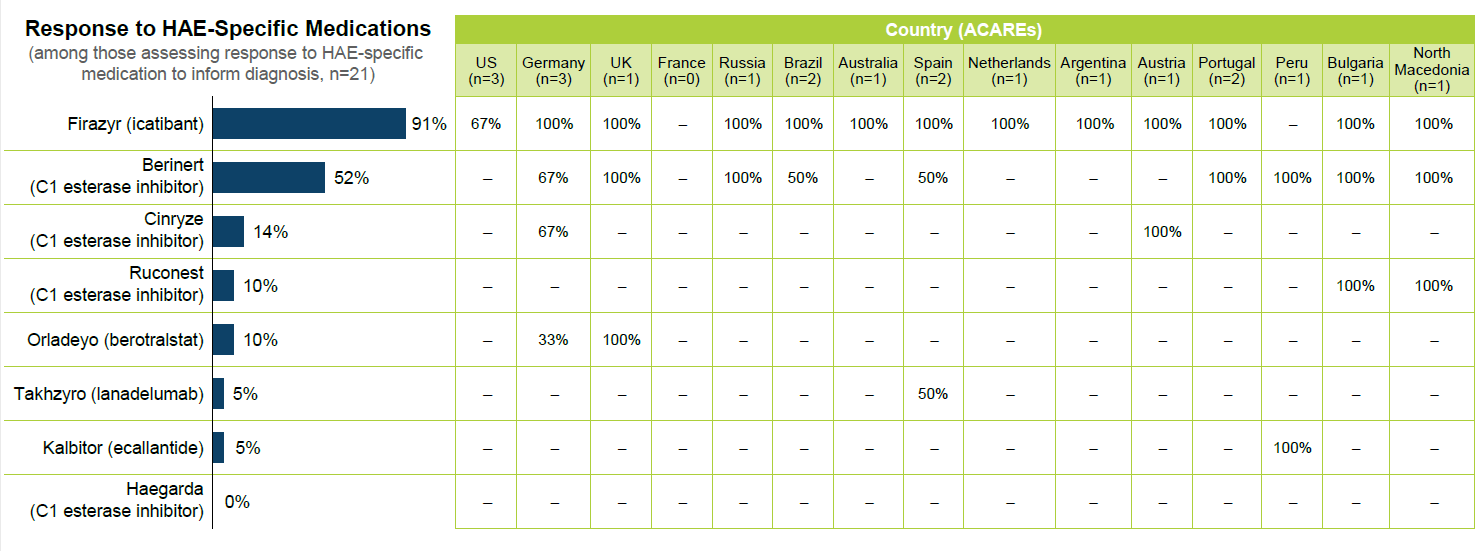


ACARE, Angioedema Center of Reference and Excellence; C1INH, C1 inhibitor; HAE, hereditary angioedema; HAE-nC1INH, HAE with normal C1INH activity.
